# Supplementary material for: Simulation of phased alerting of community first responders for cardiac arrest
Source: PLoS One. 2026 Mar 3;21(3):e0343762. doi: 10.1371/journal.pone.0343762 (PMC12956087; doi:10.1371/journal.pone.0343762)
Supplement: S1 Table — Information reported in alignment with recommended reporting items for Community First Responder (CFR) systems (10.1016/j.resuscitation.2023.110087). (PDF) [file pone.0343762.s001.pdf]

S1 Table. Information on the GoodSAM New Zealand first responder system.
Information reported in alignment with recommended reporting items for Community First Responder (CFR) systems (10.1016/j.resuscitation.2023.110087).

Category	Item	Description	The New Zealand dataset
1A: Geospatial	1: Name of the region	Specify the country, city, county, district where the system is established. Describe, whether the study includes data from the whole system or only parts of the region.	Country = New Zealand<br><br>District = National<br><br>Whole or parts of system = Whole of system
	2: Square km covered	Square km covered by the system	Approx 268,315 sqkm (ref https://doi.org/10.1016/j.resuscitation.2022.01.011)
	3: Inhabitants	Number of inhabitants of the area covered by the system in the study time	Dec 2019 population, 5,027,100 (ref https://www.stats.govt.nz/topics/population/)
	4: OHCA incidence	Number of OHCA cases per 100,000 inhabitants per year	2023/24 statistics - 134 per 100,000 https://www.stjohn.org.nz/news--info/our-performance/clinicalaudit-and-research/cardiac-arrest-annual-report/
1B: System description	1: Name of the app	What is the name of the software (app, backend system)?	GoodSAM Responder App (https://www.goodsamapp.org/cardiac)
	2: Who runs the system? Profit/Non-profit?	Which organisation is responsible for the operation of the system?	GoodSAM, https://www.goodsamapp.org/about
	3: Relevant authorities	Which authorities are responsible/supervise the system?	Hato Hone St John New Zealand
	4: AED network/database	Is the alerting system linked to an AED database? If yes, give the name (reference)	The GoodSAM AED registry, https://www.goodsamapp.org/aed

		of the AED database.	
	5: Number of FR	Give the total number of first responders who were active during the study period	5,258 (July 2019)<br><br>6,194 (July 2020)
1C: Role of dispatch centre	1: Number of emergency dispatch centres	Is more than one dispatch centre involved? Are there differences in the relevant procedures? (e.g., different indications for dispatch).	No, they are all dispatched from the New Zealand 111 call centre(s) using the same algorithms.
	2: Systems in use	Is more than one alerting system in use? Overlapping areas? Compatibility?	No
	3: Activation through emergency dispatch centre or without dispatch centre	How is the system triggered? Integrated into dispatch centre control system? Can the system be triggered without dispatch centre involvement?	Integrated into the dispatch control centre, automatically triggered by computer-aided dispatch determinants.
	4: Activation of system triggered manually/automatically	Is the system triggered automatically (if activation criteria are fulfilled)? Or is the system triggered manually by the dispatcher?	Yes triggered automatically as per Q3 above.
	5: Mission cancellation (dispatch centre)	Can the dispatch centre notify FR via the app if they cancel a mission?	Yes
	6: Mission abort (FR)	Can FR notify the dispatch centre	No, they can abort the mission but there is no notification back to dispatch.

		via the app if they decide to abort a mission?	
1D: Maturity of responder system	1: Maturity	Give some details about the maturity of the system. Is it in a pilot phase or well established?How old is the system (years)?	Well established, it was initiated in December 2017 and was still in place in June 2025
1E: Daytime	1: Times for system activation	At which times is the system active (day/night; weekday/weekend)?	Active 24/7
1F: Role allocation	1: Number of first responders (FR) alerted in a single mission	How many responders get alerted? (This does not represent the number of FR who accepted and arrived).	3 at the time of study (but 6 in 2025)
	2: Roles in the system	Describe the roles assigned to each FR by the system and the number of FR assigned to the respective role (e.g., 2 FR go to patient, 1 FR goes to AED).	There are no distinct roles.
1G: Activation criteria	1: Trigger for activation of the system	Which indications trigger the activation of the system?	02E01 ALLERGY INEFCT BRTHNG<br>02E01I ALLERGY INEFCT BRTHNG<br>02E01M ALLERGY INEFCT BRTHNG<br>09D01 ARREST INEFFECT BRTHNG<br>09E01 RESPIRATORY ARREST<br>09E02 ARREST UNCERTAIN BRTH<br>11E01 COMPLETE OBSTRUCTION<br>11E01C COMPLT OBSTRUCTN-LOLY<br>11E01F COMPLT OBSTRUCT-FOOD<br>11E01M COMPLT OBSTRUCT-LIQUID<br>11E01O COMPLT OBSTRUCT-OBJECT<br>11E01U COMPLETE OBSTRUCT-UNK<br>12D01 FIT NOT BRTHNG<br>12D01E FIT NOT BRTHNG - HX<br>14D01 DROWNING UNCON/ARREST<br>14D01D DRWN UNCON/ARREST DIVE

			14D01F DRWN UNCON/AREST-F RES<br>14D01I DROWNING ARREST ICE<br>14D01S DROWNING ARREST SCUBA<br>14D01W DROWNING ARREST-S RES<br>14E01 DRWN ARREST OUT OF WTR<br>14E01D DRWN ARREST O/W DIVE<br>14E01F DRWN ARREST O/W-F RES<br>14E01I DRWN ARREST O/W ICE<br>14E01S DRWN ARREST O/W SCUBA<br>14E01W DRWN ARREST O/W-S RES<br>31D01 UNCON AGONAL/POOR RESPS<br>31E01 UNCON/FAINT POOR RESPS<br>RESP1 CARDIAC ARREST
	2: Exclusion criteria	Describe any exclusion criteria for the activation of the system, e.g., crime scene, trauma, road traffic accidents, children	17D02 FALL ARREST<br>17D02E FALL ARREST-ENVIRO<br>17D02G FALL ARREST-GROUND<br>17D02J FALL ARREST-SUICIDE<br>17D02P FALL ARREST-PUBLIC<br>21D01M HAEM ARREST MEDICAL<br>21D01T HAEM ARREST TRAUMA<br>30D01 TRAUM INJ ARREST<br><br>We stand-down GoodSAM using a short code by our call handlers if a safety issue is known<br><br>We have specific addresses that are listed as hazardous that will be blocked from receiving a GoodSAM alert
1H: Delay time to system activation	1: Delay time	The delay time is the time from activation of the system (usually running on a server) until the first responders are being alerted through app or text message. Please describe the usual delay in your system.	Unknown
1J: Places alerted to (private, public)	1: Sites FR are dispatched to	Sites where a suspected cardiac arrest leads to an activation of the system (public places, private places)	All sites are included, both public and private.

	2: Sites FR are not dispatched to	Sites where a suspected cardiac arrest does not lead to an activation of the system (e.g. care homes)	There are no exclusions
1K: Characteristics of FRs	1: FR groups	Which groups are serving as FR? If possible, give the number/percentage of firemen, policemen, nurses or other groups involved into the program.	Emergency Medical Services Personnel and Fire Service ~30%<br><br>Members of the public ~30%<br><br>Registered health professionals ~30%
	2: On duty/off duty	Are the FR usually available when they are on duty or off duty?	We do not use an on/off duty function. Responders can make themselves unavailable by choosing not to override the silent mode on their phone.
	3: FR duties	Describe the functions of the FR.	CPR and AED use.
