# Supplementary material for: Simulation of phased alerting of community first responders for cardiac arrest
Source: PLoS One. 2026 Mar 3;21(3):e0343762. doi: 10.1371/journal.pone.0343762 (PMC12956087; doi:10.1371/journal.pone.0343762)
Supplement: Attachment — Submitted filename: Response to Reviewers.pdf [file pone.0343762.s003.pdf]

Response to Reviewers

Reviewer #1:

Thank you for your evaluation of our manuscript. We appreciate your constructive comments, criticisms and suggestions.

The authors present the results of a simulation-based study that investigates the impact of different phased alerting strategies on Community First Responder (CFR) systems.

This study offers an interesting approach to addressing this relevant issue, and the findings could potentially influence CFR alerting strategies, albeit within the outlined limitations. Although the simulations are complex and challenging to reproduce, the detailed explanation of methods, including the parameters and metrics, ensures that the derivation of the results as well as the conclusions are clear and understandable.

A key question regarding the relevance of this study is the uncertainty regarding the impact of unnecessary alarms on the motivation of first responders. While an increase in the number of registered responders may lead to more redundant arrivals depending on the alerting strategy, it also allows these arrivals to be distributed across more individuals, potentially reducing the negative effect on motivation. In regions with a relatively low density of first responders, however, the findings presented in the manuscript become even more significant.

In their submission, the authors provided a comprehensive explanation of why the data on which the study is based is not publicly available and that it can be viewed on request.

Some minor adaptions are recommended:

Give a definition of the abbreviation KPI the first time it is used.
Done.

I would recommend omitting the calculation of the ratios in lines 36 and 37 of the abstract and simply presenting the results as 11% and 137%.
Done.

Provide a y-axis label in the left panel of Figure 2.
We added the label “Number of alerts” to the axis.

Consider creating a comprehensive figure that graphically illustrates the processes and

scenarios analyzed. This could help make the results of the simulations more tangible for the reader.

Thank you for this suggestion. We have included a multi-panel plot that graphically illustrates the processes and scenarios:

- Panel 1 shows the typical response process for OHCA in the presence of volunteer responders.
- Panel 2 shows the design of the simulation, including the input and output data.
- Panel 3 shows how the simulation model is used to compare the different dispatching policies.

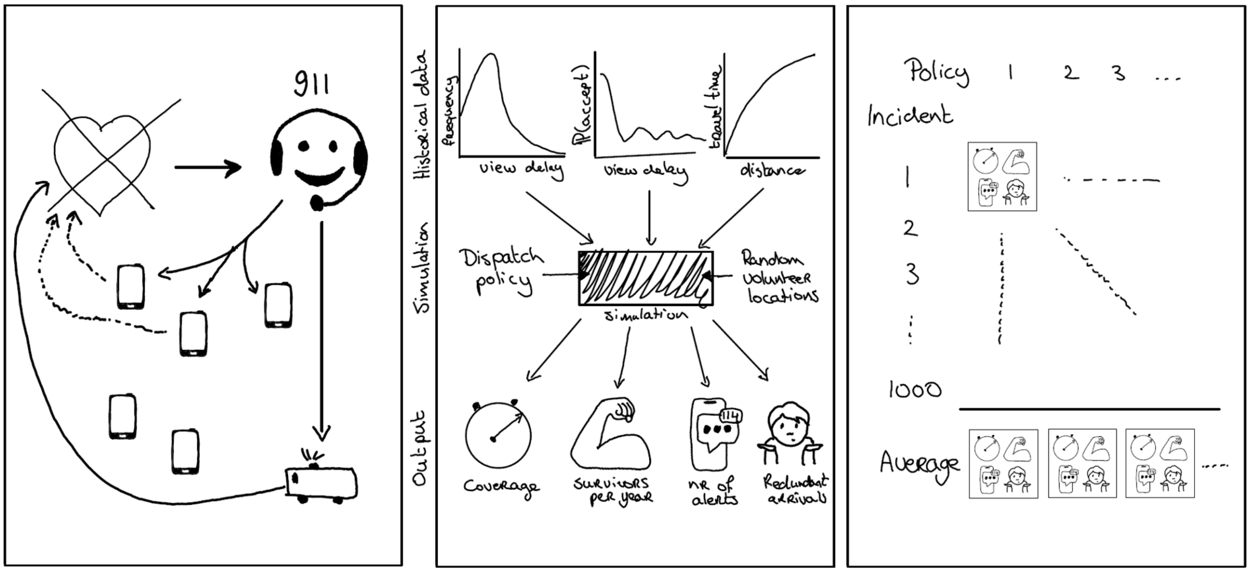

Reviewer #2:

Thank you for the opportunity to review the manuscript entitled „Phased alerting of community first responders for cardiac arrest“ by van den Berg and colleagues. The authors aimed to compare different alert policies in the first responder system GoodSam via simulation. The authors based their assumptions for the simulation partly on historical data from real GoodSam alerts in New Zealand. The data extraction was performed on January 15th, 2021. The manuscript explores a topic that should be of interest to the readers of PLOS ONE.

Unfortunately, I have to raise several major concerns regarding the manuscript. Multiple statements are overstated and do not follow current reporting standards for first responder systems. Furthermore, the transferability of the results is highly doubtful.

Major comments:

Introduction

1. Lines 51-56: It is difficult to describe the simple use of phased alerts as “complex alerting”. Several systems today already take into account the expected arrival time of the emergency services and the current traffic situation, as well as the means of transport of the first responder. The reader should be made aware that more complex alert algorithms are already possible. The study and the results refer to the GoodSam system only. The transferability of the results to other systems is extremely limited.

Thanks for pointing out that more complex alert algorithms are already possible. We were aware of this, but acknowledge that that did not come across well enough in our text. We updated the introduction (second paragraph) and no longer claim that phased alerts alone qualify as ‘complex’.

Regarding the GoodSAM system, it currently offers settings for user-defined batch size and time lag between batches. While already somewhat flexible, one can wonder whether this framework uses phased alerting to its full potential. This may also be true of other systems. Our work investigates policies that are *not* restricted by the current GoodSAM setup, and would for many CFR systems likely require only a small software change. Please view our work as indicating the potential of phased alerting algorithms, if used well. We have modified the text in 1.2 to clarify this perspective.

2. Lines 58-63: Volunteer fatigue can theoretically pose a problem in a first responder system. However, there is no study that has ever shown that volunteer fatigue occurs or that there are limits to, for example, false alarms or arriving at the scene with other responders, which trigger volunteer fatigue. Or could the authors find any evidence of volunteer fatigue

in their own historical data? If so, how did this affect the historical data on which your model is based?

Thank you for pointing this out. Indeed, there is no study on volunteer fatigue and hence we recommend for one in our Future Research section (4.2). The absence of such studies is also what led us to refrain from recommending a specific dispatch policy, instead presenting a list of options from which a CFR system manager can choose.

The historical data did not allow us to detect volunteer fatigue (nor the absence thereof) as the system only sent out up to 3 alerts per patient, and with an acceptance ratio around 19%, that gives almost always 0 redundant arrivals.
We also empirically investigated volunteer fatigue based on number of alerts, but we could not detect a positive correlation there either, which we believe to be caused by the low number of alerts per person in the GoodSAM system: in our entire database there were only 10 volunteers who received more than 20 alerts in any given year (and nobody more than 35).

Despite the above, we still believe volunteer fatigue is real, see for example the “Cry Wolf” [1] phenomenon, which refers to the tendency of individuals to ignore or delay responses to repeated warnings—such as alarms or alerts—when prior warnings have been perceived as false or irrelevant.

The current practice with GoodSAM in Auckland is to send limited alerts, which suggests a goal of avoiding volunteer fatigue, since otherwise the protocol would be to just dispatch everyone within a reasonable range.

3. In my personal opinion, the chance to trigger volunteer fatique is a lot greater, if volunteers arrive after EMS than multiple first responders arriving before EMS on scene. How could this be implemented in your simulation?

Many thanks for this very interesting idea. It matches our own experience as a CFR. It could be implemented by simply removing the counting of previously arrived volunteers as redundant. Then the redundant arrivals will simply reflect the count of those volunteers that arrive after EMS.

While the above in principle answers your question, please allow us to elaborate. If one did wish to do an experiment in this direction, we have a few suggestions on how to improve it:

---

[1] Breznitz, S. (2013). *Cry wolf: The psychology of false alarms*. Psychology Press.

- In our current experiments, EMS response time is a fixed number. The new EMS-centric context makes it more important to model EMS arrival accurately, therefore we would recommend obtaining a good estimate of the EMS response times across the city.
    - Preferably not just averages, but the whole distribution (histogram).
    - This can either come from historical data, or if absent, from a separate simulation using one of the available open-source ambulance simulation packages (see [2] for an example where this is done for CFR modeling). In the Monte Carlo simulation, one can then sample from this histogram.
- Perhaps one would also want to analyze a different list of policies. This is because the 'keep active' policies are mainly inspired by volunteers affecting each other's fatigue.

4. This is not the first study to evaluate different alerting policies in a community first responder system (also refer to: 10.1007/s10049-024-01395-2). This might be the first study to evaluate alerting policies in GoodSam. Please clarify.

We apologize for having missed this paper. If we understood the German correctly, this app asks the volunteers their travel mode before deciding who does what. This is an impressive feature, that as far as we know, is at the forefront of what CFR systems facilitate worldwide. We cited the paper in the Discussion section.

5. Please refer to the reporting standard for describing first responder systems, smartphone alerting systems, and AED networks (10.1016/j.resuscitation.2023.110087). Several core parameters are missing in your manuscript and need to be included to get an overview of the first responder system in New Zealand.

Thanks for pointing out these reporting standards. We added as much as we could in a table in our appendix. We also added a reference to this appendix in Section 2.4. We regret not being able to provide *all* information, but these recommendations are really extensive and it seems common in the CFR literature to not fill in all of them, see for example, [3] [4].

Methods

---

[2] van den Berg, P. L., Henderson, S. G., Jagtenberg, C. J., & Li, H. (2025). Modeling the impact of community first responders. *Management Science*, *71*(2), 992-1008.

[3] Ganter, J., Busch, HJ, Trummer, G., Schmitz, D., Pooth, JS, Steuber, T., ... & Müller, MP (2024). From smartphone-based first responder alerting to a "life-saving system." *Emergency + Rescue Medicine* , 1-9.

[4] de Greef, B., Genbrugge, C., Verma, S., Medic, G., Maurer, J., Kooy, T. A., ... & Sabbe, M. (2025). Cost-effectiveness of a community first responder system for out-of-hospital cardiac arrest in Belgium. *Open Heart*, *12*(1).

6. Lines 102-103: Is it correct, that given “that no alerts are sent out after a volunteer accepts an alert”. Most cases would only have one first responder on scene? To perform high quality chest compressions, current guidelines recommend changing ever two minutes. Therefore, at least two responders on scene are needed. The rate of alarms with at least 2 responders on scene should be included as an KPI in your model.

Thank you for your question. Yes, it is correct that no alerts are sent out after a volunteer accepts. This is both the case in the GoodSAM system as well as in our simulations. However, note that there may be other outstanding alerts at that moment, and those volunteers may still arrive at the patient.

We appreciate your point that a second responder can be beneficial to assist the first in performing high-quality chest compressions. As you requested, we added the fraction of alarms with at least 2 responders on scene as a KPI, which is reported in an extra column in Table 1-3. You will note that numbers in other columns have also changed in the revision. This is because we ran all simulations again to produce the updated results, and if you look closely you will see that these are *very minor changes* that merely reflect the variation between simulation runs.

7. Lines 102-103: “or 10 minutes have passed after GoodSAM activation”. Since the expected time of EMS arrival seems to be unavailable in GoodSam and an EMS arrival time of 13 minutes after the initial alarm is assumed (compare line 166), it is surprising that the authors chose 10 minutes as a cut-off to stop alerting. Taking into account the authors’ historical data (Figure 3b), a first responder alerted after 9 or 10 minutes would need to be within 200 m of the patient to arrive within 3 minutes (before the EMS) and to have an impact on patient survival. Please also refer to comment #3 in this context. Also: Since the authors assume a witness and triage delay of 3 minutes in all cases (Lines 168-170), the EMS would already have arrived, when alerting a first responder after 10 minutes.

Thank you for your attention to detail in this matter. Indeed, we use an T_EMS of 13 minutes and witness plus triage delay of 3 minutes, making 10 minutes exactly the moment after which volunteers are guaranteed to arrive after EMS.

This leaves us to discuss alerts just before the 10-minute mark. Your Comment #3 is about triggering fatigue when a volunteer arrives after EMS, and indeed, alerting just before 10 minutes leaves little time for a responder to arrive prior to EMS. Our cut-off time did not depend on the distance between volunteer and patient, and in that light, any cut-off time other than 10 minutes appears arbitrary.

8. Line 122-123: Please clarify what GPS radius was used to measure the CFR's arrival at

the scene. The chosen GPS radius has a significant impact on the accuracy of your data (10.1080/10903127.2021.1983094).

A GPS location within 50 meters of the scene is recorded as on-scene. Responders can also manually press a button to generate an on-scene timestamp. We have included this information in the description of the data in 2.4.

9. Lines 123-132: Please provide further information on your historical data as recommended by the reporting standard (see comment #5). For example, activation rate, response rate and arrival before EMS rate are missing core parameters.

We now provide these values in the appendix.

10. Lines 170-175: The calculated survival probability, which is included as a KPI, is a very poor estimation. Please provide data on the observed survival in the observation period given the current first responder system alert policy. How does this data compare to the calculated survival probability?

Survival prediction is not the primary aim of this paper, and this data was not available to the researchers at the time of this analysis. Moreover, due to the low survival from cardiac arrest, this would require significant longitudinal data and include adjustment for confounding using regression or similar.

We'd like to emphasize our idea of comparing similar scenarios (i.e., patients with volunteer response, just different response times) under different conditions. The main insight is the trade-off between volunteer fatigue and provided care. Any consistent over- or underestimation in the model tends to affect both scenarios similarly, reducing the impact of bias on the comparison. While a different survival curve would give different numbers, we believe the insight and the trade-off do not too heavily depend on the specific survival curve.

11. Lines 173-175 and 166: It is surprising that the authors chose to rely on data from the ministry of health or "EMS targets" rather than the existing OHCA registry report from the time the historical data was extracted (e.g.: https://www.resus.org.nz/assets/OHCA_All_NZ_March_2022_HQ.pdf). I recommend relying on the available real-world data and to **redo** the analysis. The current data basis for the basic assumptions of the simulation is simply not transferable. Please explain.

Thank you for pointing out this document. Page 4 reports a median EMS response time of 8 minutes for urban and 12 minutes for rural regions. Our used value matches the latter,

which we describe as 13 minutes (equal to their reported median of 12 because we include a 1-minute witness delay in the count). We added this to our paper on line 162.

Conclusion and Abstract

12. Line 38: Please rewrite the following statement: Monte Carlo simulation is suitable to predict how changing a CFR alert policy affects survival and volunteer fatigue. None has been shown in your manuscript. The transferability of your results is highly doubtful.

Thank you for pointing out that this statement was too strong, we softened our claim.

13. Lines 30-33: Up to today, we are unfortunately still lacking sufficient evidence to claim that first responder systems improve survival after OHCA. So far only improved rates of bystander CPR could be proven with no effect on survival. Please also refer to the current AHA or ERC guidelines or ILCOR for further information. Your results are therefore completely overstated, especially in light of comment #11.

Thank you for pointing out that this statement was too strong, we softened our claims throughout the paper. We also added a paragraph under Limitations.

14. In your discussion: Since you acknowledge that volunteer density may vary regionally and depending on the time of day, how should CFR system managers estimate the volunteer density in advance? Please explain. In my personal opinion, a “smart” alerting system needs to check the CFR density within its alerting algorithm.

The number of volunteers in the circle is known in real time, so the policy for alerting can be chosen based on that information. Our simulations shed light on which policies should be considered for which numbers of volunteers.

In a separate methodological paper, we explore the potential benefit of dynamically choosing the dispatch policy based on real-time information. This includes, but is not limited to, the number of volunteers within the circle. This paper is written for a mathematical audience as it involves the development of real-time optimization techniques using Machine Learning and Dynamic Programming.

Minor comments:

15. Please include „GoodSam“ and „Simulation“ in your title to clarify the study content.
Thanks for your suggestions. We added “Simulation of” at the start of the title and agree that this is an improvement. We are, however, a little hesitant about adding ‘GoodSAM’ to the title. Even though we used GoodSAM data for certain elements, we still believe our insights

are more generic than that. We observe just a few examples in the literature where the name of the CFR system is in the title of the paper, and these are for retrospective studies where the volunteers participated in a post-alert survey. This appears much more system-dependent than our work.

16. Lines 113/114: Please use n1 and n2 to clarify which n can have what value in lines 107-112.
Thank you for your suggestion. We have modified this in the paper.

17. Line 196: x is the distance in meters? Please clarify.
You are correct. The distance is in meters, as mentioned in line 196.

18. Figure 3b: y axis is missing units. Please clarify.
Done
