# Supplementary material for: Simulation of phased alerting of community first responders for cardiac arrest
Source: PLoS One. 2026 Mar 3;21(3):e0343762. doi: 10.1371/journal.pone.0343762 (PMC12956087; doi:10.1371/journal.pone.0343762)
Supplement: Attachment — Submitted filename: Response_to_Reviewers_auresp_2.pdf [file pone.0343762.s004.pdf]

Response to Reviewers

Reviewer #1:

Thank you for your positive evaluation of our revised manuscript.

The only remaining remark concerns the optional graphical illustration of the study design, which in its current form adds little value and may be omitted. Overall, I consider the manuscript suitable for publication.

We have followed your suggestion and have omitted the graphical illustration of the study design (Figure 2 in the previous version) and have updated all references to figures in the manuscript accordingly.
